# Supplementary figures and images for: Prediction of HIV-1 protease resistance using genotypic, phenotypic, and molecular information with artificial neural networks
Source: PeerJ. 2023 Mar 21;11:e14987. doi: 10.7717/peerj.14987 (PMC10038082; doi:10.7717/peerj.14987)

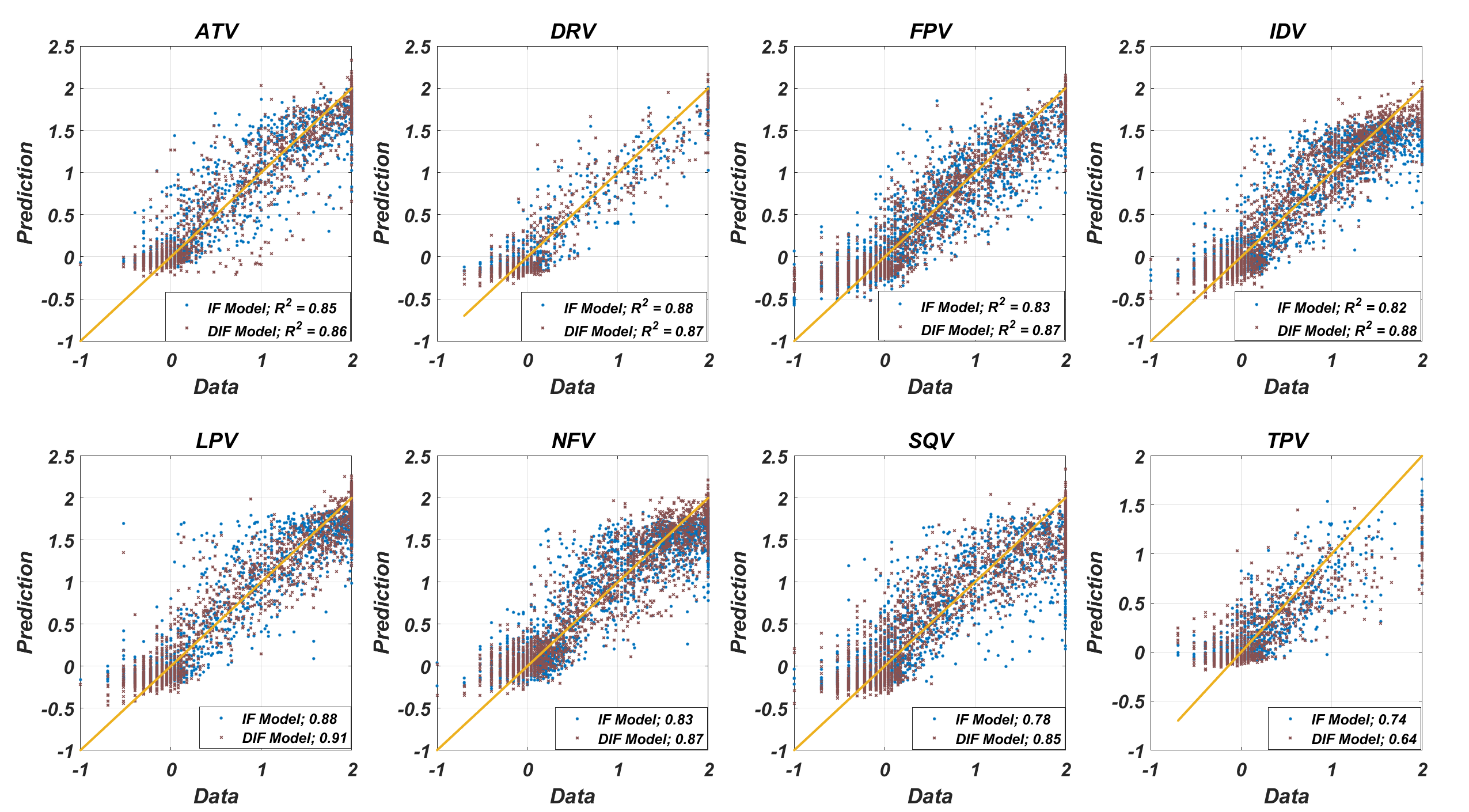

Supplement: Figure S1 [file peerj-11-14987-s002.png]

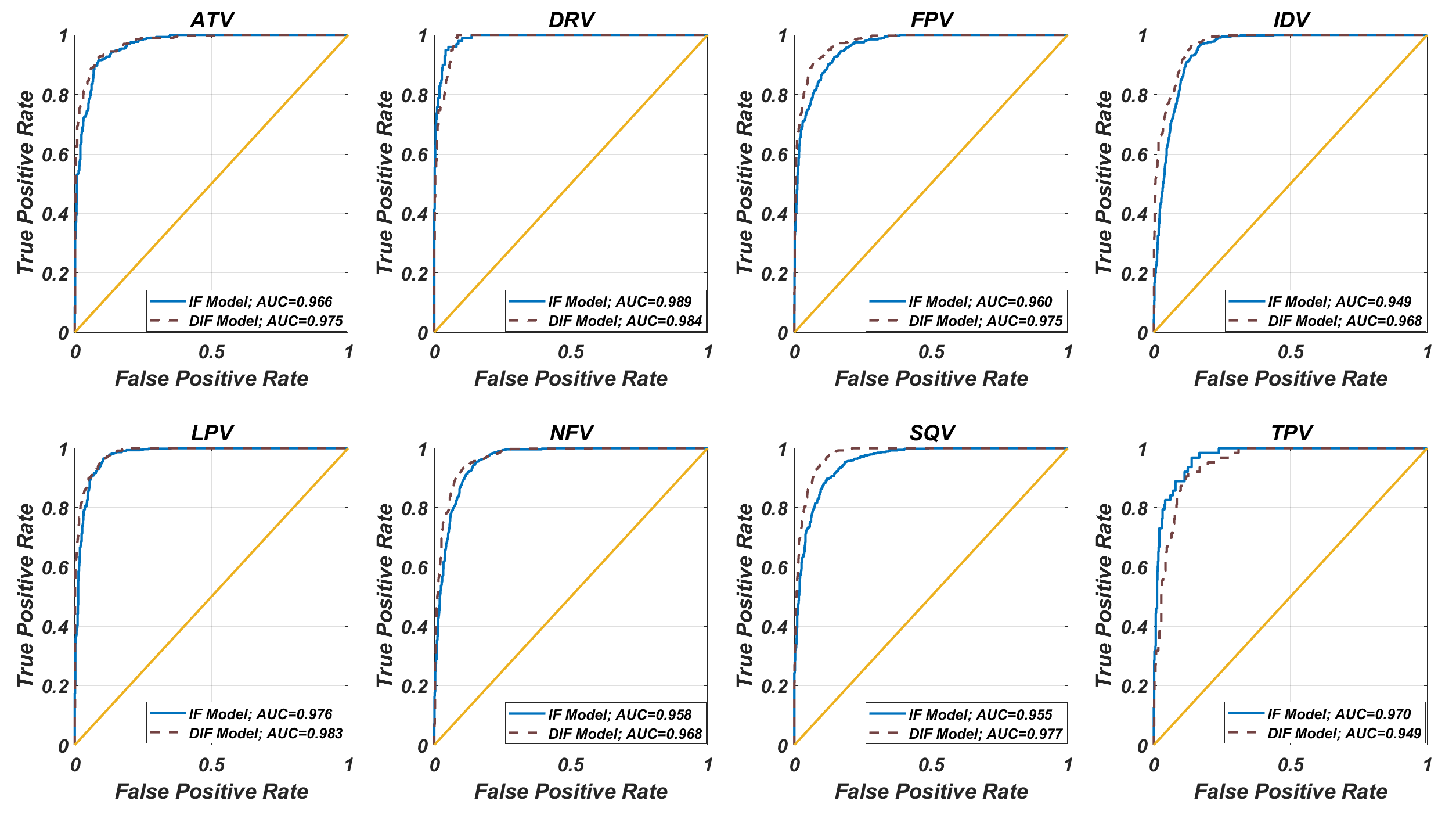

Supplement: Figure S2 — For each inhibitor, IF (isolate-fold-change) models are trained to predict fold-change values based on the isolate information. A DIF (drug-isolate-fold-change) model is trained using all the inhibitor data to predict fold-change values from isolate and molecule descriptors. For the training procedure, the ANN architecture with an ensemble learning method has been utilized, as detailed in the Materials and Methods section. The threshold fold change value for the categorization of resistant and susceptible patterns is set to 3, and the AUC values for the associated ROC curves of both models are shown. [file peerj-11-14987-s003.png]
