# Supplementary material for: Prediction of HIV-1 protease resistance using genotypic, phenotypic, and molecular information with artificial neural networks
Source: PeerJ. 2023 Mar 21;11:e14987. doi: 10.7717/peerj.14987 (PMC10038082; doi:10.7717/peerj.14987)
Supplement: Table S1 — IF (isolate-fold-change)-based models are trained for each inhibitor to predict fold-change values from the given isolate information. DIF (drug-isolate-fold-change)-based model is trained using all the data of the inhibitors to predict fold-change values from the descriptors of both isolates and molecules. Accuracy, sensitivity, and specificity values represent the rate of true predictions, true positive rate, and true negative rate, respectively. The threshold fold change value of 3 is chosen for classifying resistant and susceptible patterns. [file peerj-11-14987-s004.docx]

**Table S1.** Accuracy, sensitivity, specificity, and MCC values of the IF-based ANN models^a^ and DIF-based ANN models^b^ in classifying the resistant and susceptible patterns for each ARVs^c,d^.

| Metric | Model | ATV | DRV | FPV | IDV | LPV | NFV | SQV | TPV |
| --- | --- | --- | --- | --- | --- | --- | --- | --- | --- |
| Accuracy | IF | 0.912 | 0.953 | 0.907 | 0.888 | 0.919 | 0.893 | 0.890 | 0.958 |
|  | DIF | 0.917 | 0.941 | 0.927 | 0.898 | 0.924 | 0.912 | 0.925 | 0.943 |
| Sensitivity | IF | 0.857 | 0.876 | 0.823 | 0.783 | 0.869 | 0.845 | 0.829 | 0.755 |
|  | DIF | 0.872 | 0.817 | 0.850 | 0.816 | 0.894 | 0.894 | 0.865 | 0.762 |
| Specificity | IF | 0.946 | 0.964 | 0.928 | 0.944 | 0.956 | 0.933 | 0.913 | 0.970 |
|  | DIF | 0.944 | 0.961 | 0.948 | 0.940 | 0.944 | 0.925 | 0.949 | 0.947 |
| MCC | IF | 0.812 | 0.804 | 0.724 | 0.750 | 0.834 | 0.785 | 0.728 | 0.645 |
|  | DIF | 0.821 | 0.758 | 0.788 | 0.768 | 0.841 | 0.820 | 0.817 | 0.419 |

^a^ IF (isolate-fold-change)-based models are trained for each inhibitor to predict fold-change values from the given isolate information.

^b^DIF (drug-isolate-fold-change)-based model is trained using all the data of the inhibitors to predict fold-change values from the descriptors of both isolates and molecules.

^c^Accuracy, sensitivity, and specificity values represent the rate of true predictions, true positive rate, and true negative rate, respectively.

^d^ The threshold fold change value of 3 is chosen for classifying resistant and susceptible patterns.
